# Methodology for Eddy Current Losses Calculation in Linear Variable Differential Transformers (LVDTs)

**DOI:** 10.3390/s23041760

**Published:** 2023-02-04

**Authors:** Ana Drandić, Stjepan Frljić, Bojan Trkulja

**Affiliations:** Faculty of Electrical Engineering and Computing, University of Zagreb, Unska 3, 10000 Zagreb, Croatia

**Keywords:** LVDT, eddy current losses, numerical simulation, FEM

## Abstract

Linear variable differential transformer (LVDT) is a commonly used linear displacement sensor because of its good measurement characteristics. When using laminated ferromagnetic cores in LVDTs, it is very important to take eddy currents into the account during design phase of the sensor. Particularity of the open-type core means that the eddy currents induced by the stray magnetic flux that flow in large loops tangential to the lamination surfaces take on significant values. Due to the open-type core a typical LVDT has, depending on the core material, it is, therefore, very important to take eddy currents into the account when designing the sensor. This paper’s goal is to present a methodology for calculating LVDT eddy current losses that can be applied to LVDT design in order to optimize the dimensions and help with selection of materials of the LVDTs, in order to achieve the highest measurement accuracy. Presented approach using an AτA-formulation with elimination of redundant degrees of freedom exhibits rapid convergence. In order to calculate the relationship between eddy current losses and core displacement, frequency, and material characteristics, a number of 3D finite element method (FEM) simulations was performed. Analysis of the obtained results using presented methodology for eddy current losses calculation in LVDTs enables the designer optimize the design of the LVDT.

## 1. Introduction

Different industries often use magnetic position sensors due to their robustness, reliableness, and cheapness. New designs of transformer position sensors are constantly developed and analysed to improve their industrial applicability. Finite element method (FEM) is often used for modeling and analysis of new sensor design [1,2,3,4,5,6,7]. In [1], the electromagnetic behavior of an LVDT sensor in the presence of magnetic interference is modeled using FEM, and the quality of the simulation is verified through comparisons with experimental results. In order to optimize the sensor design, [2] uses FEM to study the magnetic field distribution of a flat-type magnetic position sensor. In [3], the electromagnetic behavior of differential inductive displacement sensors is modelled using FEM, and the parameters impacting the sensor’s time drift stability are analysed. FEM is utilized in [4] to model the electromagnetic behavior of PCB-based rotary-inductive position sensors, and in [5] to model the electromagnetic behavior of inductive displacement sensors with large range and nanoscale resolution. In [6], FEM is used to simulate the electromagnetic behavior of LVDT sensors in order to improve sensor design. In [7], the electromagnetic behavior of LVDT sensors is also simulated using FEM in order to examine the effects of process and material parameters on the sensor output characteristics. A good topical review of magnetic position sensors can be found in [8].

Linear variable differential transformer (LVDT) is a widely used linear displacement sensor. It is known for its robust structure, high linearity, high precision, and contactless nature [2,9]. LVTD is used for manufacturing, control, and scientific applications. The conventional LVDT design is of a cylindrical structure that includes a core, one primary, and two secondary windings [2,10,11]. The core is usually made of a magnetic material, such as ferrites or soft magnetic materials.

LVDTs, like other inductive sensors, have some limitations. Limitations include limited linear range, stray capacitance effect, electromagnetic interference, and core loss. That is why various studies, techniques for improvement, as well as novel LVDT designs for specific, or broader, applications have been investigated [10,12,13,14,15,16]. In [15], a compact LVDT for reactor experiment application was designed using analytical expressions, without the consideration of eddy current losses. In [1], the study of effect of magnetic interference on the LVDT sensor was made. Modified design of LVDT is also proposed in [10], with the study of the effect of the external magnetic field on the secondary using FEM-based software. Another modified design is presented in [2], where a flat LVDT sensor is introduced with external armature made of solid iron and steel laminations. Minimization of the the error of the LVDT output signal due to the temperature effect is presented in [17]. In [2], effects of the induced eddy currents in the laminations are evident for frequency of 400 Hz. Inductive displacement sensors are analysed in [5], where core loss influence on the quality factor is examined. A study of magnetic core materials used in LVDT is presented in [18]. There, a correlation between eddy current effects and material sensitivity and linearity can be seen, emphasizing the need of eddy current analysis for LVDTs.

As previously mentioned, the cores of LVDTs can be made of different magnetic materials. In [19,20], the use of FE-rich amorphous wire and glass-covered amorphous wires as active core is presented. Additionally, ferrite is sometimes used to make the cores. However, ferrite has some drawbacks, such as high brittleness and low strength when geometric changes are needed. The analysis of core losses is necessary due to the use of various materials. Core losses can be categorized into hysteresis losses and eddy current losses. Hysteresis losses are proportional to the flux density and are represented by the area in the hysteresis loop. When ferromagnetic cores are used, eddy current losses can be an important consideration. Eddy currents are circulating currents that are induced in a conductor when it is subjected to a changing magnetic field. The flux moving through the core causes eddy currents to form, and losses resulting from those currents increase with frequency [18]. In order to decrease the losses, laminated cores are used. In LVDT, the laminated core is typically made of a stack of thin sheets of a magnetic material. The thin laminations help to reduce eddy current losses by breaking up the continuity of the core and reducing the size of the circulating currents. The core material and the thickness of the laminations also play an important role in reducing eddy current losses. Investigation in [5] has shown that eddy current losses are small enough that the core does not have to be laminated if the conductivity of the core is less than $1\times 10^{4}$ S/m. For higher conductivities, laminations must be used in order to reduce the eddy current loss. Otherwise, the sensors quality factor and possible excitation frequency will be negatively affected due to eddy current losses. Therefore, due to their effects on the sensor accuracy, eddy current losses must, therefore, be considered when designing the sensor, as well. Additionally, it is important to take into account the frequency range in which the LVDT will be operated. The frequency response of an LVDT is determined by the design and construction of the transformer and the electronic signal conditioning circuit. It is important to note that LVDTs are sensitive to AC excitation frequency, the measurement frequency range will vary depending on the specific device and its design. In general, the eddy current losses are an important consideration in LVDT design but can be minimized through proper core design and materials selection.

Computational algorithms used for LVDTs mostly fail to take account of eddy-current effects [21]. Despite the fact that numerous studies suggest that a comprehensive consideration of core losses, particularly eddy current losses, should be completed, the quantification of those losses is missing [2,5,18,21]. To the best of the authors’ knowledge, there are no scientific papers addressing specifically the eddy current losses in laminated cores of LVDTs. Therefore, it is the purpose of this paper to introduce the methodology for eddy current losses calculation of LVDTs that can be used during the design of LVDTs in order to optimize the dimensions of the LVDTs and help when selecting core materials, all for the goal of achieving the best measurement characteristics of LVDTs.

Analysis of eddy current problems in laminated cores is of great interest and it has been the topic of various papers [22,23,24,25]. However, calculation of eddy current losses requires taking into account differences between the open-type core and the closed-type core. Core of the conventionally designed LVDT is an open-core type [26,27]. Compared to a closed-type core, where the magnetic flux is closed mostly through the core, the of the magnetic flux of the open-type core passes through core/air interface and majority of the flux is dissipated perpendicularly to the lamination surfaces. As a result, the eddy currents generated by the stray magnetic flux that flow in wide loops tangential to the lamination surfaces can acquire considerable values. Moreover, since the problem domain of laminated medium of the represents a region of heterogeneous material, a very dense finite-element mesh is necessary inside the core region in order to account for eddy currents and material heterogeneity. Due to the lengthy simulation and the high demands on the computer’s working memory, such a problem is almost impossible to solve. Therefore, homogenization is frequently employed [22,23]. The particular geometry of open-type cores, along with the use of laminations, means that commercial software based on general techniques is usually not well adapted for analysis of eddy current losses. Overall, there is scarce research on the problem of eddy currents in open-type cores. A multi-scale approach that includes the modeling of eddy currents is presented in [28], while a two-step method for total eddy currents calculation in an open-type laminated core is presented in [26]. Research conducted in [26] shows the advantages of the use of an AτA-formulation compared to AVA formulation when calculating eddy-current losses in open-type cores.

The methodology presented in this paper for the eddy current losses calculation in LVDT core is based on a 3D finite element method (FEM) approach based on a weak AτA-formulation. A similar approach was used in [26], but in this paper a novel and improved approach for the elimination of redundant degrees of freedom which results in improved speed of convergence of the calculation. Results of the approach using a weak AτA-formulation with and without edge elements will be presented as validation of the acquired results and evidence of method advancement. The presented methodology is valid in linear cases, for LVDTs designed to work in the frequency range 50 Hz to 500 Hz, so the focus is on applications in the lower frequency range. The results obtained using the presented methodology are analysed using a LVDT model. The contribution of this paper is the demonstration of the correlation between eddy current losses and core displacement, frequency, and material properties by using a novel approach for the calculation of eddy current losses based on the weak AτA-formulation. Even through a conventional LVDT model was used for the analysis of eddy current losses, presented methodology can easily be used for calculation of losses in modified LVDT designs in which laminations in core or armature exist.

This paper is organized as follows: Section 2 discusses LVDT design, gives an overview of eddy currents, and presents the robust method for fast calculation of eddy current losses. Analysis of the results achieved by using said method and validation, along with discussion are presented in Section 2. In Section 4, the conclusion is made.

## 2. Theoretical Overview

In this section, a brief overview of LVDT design and principles is given, as well as an introduction in eddy currents in LVDT core. Then, the methodology for eddy current losses calculation is presented.

### 2.1. LVDT Design

**Figure 1:**
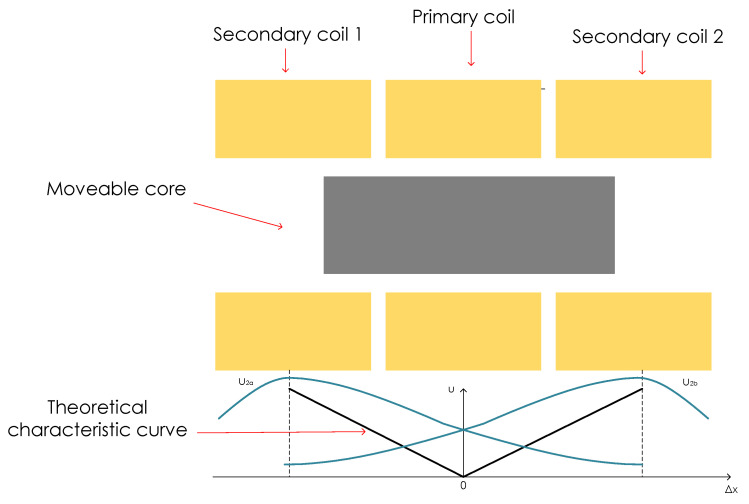
Structure of a LVDT with characteristic curve of output voltage.

The main LVDT components are a primary coil, two secondary coils symmetrically spaced with respect to the primary coil, and magnetic core that is moveable, as depicted in Figure 1. Primary coil is the excitation coil, and secondary coils are the pickup coils of the sensor. Since the structure is symmetrical, core in the middle (centre) position has same length portions embraced by the pickup coils. This, and the fact that the secondary coils are connected in reversed series, giving the zero-voltage output for the centre position of the core when alternating current of appropriate amplitude and frequency is applied to primary winding. The LVDTs output is the differential voltage between the two secondary windings. Moving the core along the axis means that one of the secondary windings will embrace more, and the other less of the core. This means that the flux linkage between the windings will change according to core displacement from the centre position. This will result in the increase in voltage in one secondary coil, while the voltage in the other secondary coil will decrease. Plot of output voltage is represented by the theoretical characteristic curve shown in Figure 1.

As already mentioned, different materials are used for the manufacture of LVDT cores. Depending on the material used it is important to investigate the potential drawbacks it brings to the designed LVDT. Due to the open-type laminated core of LVDTs, when using ferromagnetic materials the eddy currents generated by the stray magnetic flux that flow in wide loops tangential to the lamination surfaces can acquire considerable values. Therefore, when it comes to the cores made with ferromagnetic materials, eddy current effects, including losses due to eddy currents, should be taken into account. In the next subsection, eddy current losses calculation method, based on a weak AτA-formulation is presented and explained.

### 2.2. Eddy Current Losses Calculation Method

**Figure 2:**
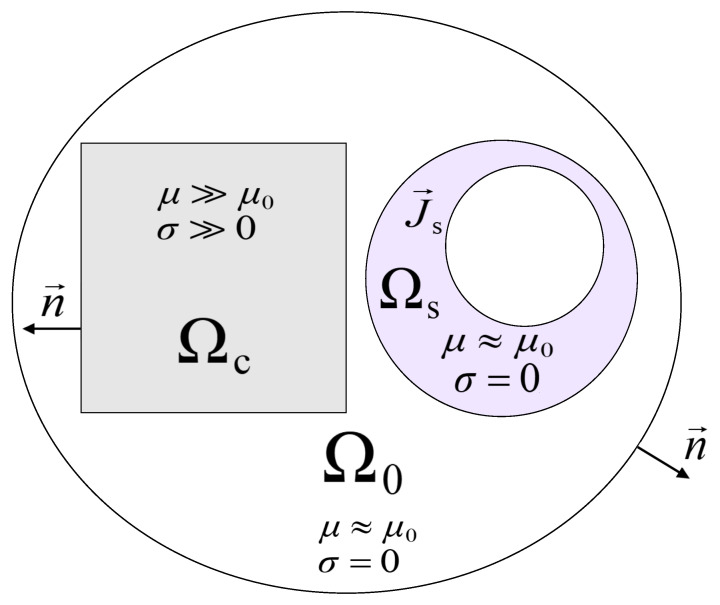
Definition of problem domain.

Defining the problem domain is the initial step in calculating the eddy currents. Figure 2 illustrates the three regions that make up the problem domain. Region $\Omega_{c}$ stands in for the core region, $\Omega_{0}$ for the air region, and $\Omega_{s}$ for the winding region. Vector ${\vec{J}}_{s}$ stands for the source current flowing through the winding region. As was already noted, the core region is made of magnetic material, and the linear properties of that material are assumed in the presented work.

Assuming a low-frequency range, the set of Maxwell’s equations, in which $\vec{B}$ is the vector field of magnetic induction, and $\vec{J}$ is the vector field of eddy current density, for the core region is:

(1) $$\nabla \times \rho \vec{J}=-j\omega \vec{B}$$

(2) $$\nabla \times \nu \vec{B}=\vec{J}+{\vec{J}}_{s}$$

(3) $$\nabla \cdot \vec{B}=0$$

(4) $$\nabla \cdot \vec{J}=0$$

where material electrical resistivity and its magnetic reluctivity are represented by ρ and ν, respectively, and ${\vec{J}}_{s}$ is the source current density. It is assumed that the source current density in the core region is zero.

The air and winding regions are both classified as non-magnetic and electrically non-conductive since only the core region is important for calculating eddy current losses. As a result, the Maxwell’s equations that represent those regions are:

(5) $$\nabla \times \nu_{0}\vec{B}={\vec{J}}_{s}$$

(6) $$\nabla \cdot {\vec{J}}_{s}=0$$

where $\nu_{0}$ is the magnetic reluctivity of vacuum.

Calculating eddy current losses in impacted areas is possible after the problem domains are identified. The outer laminations of an LVDT’s core are where eddy currents are most prevalent. The perpendicular component of the magnetic induction, when seen in relation to the laminations, is what primarily induces eddy currents in outer laminations. The tangential component of the magnetic induction is primarily responsible for inducing eddy currents in the inner laminations (relative to the laminations). The normal and tangential components of the magnetic induction and eddy current density should, therefore, be separated, as this would be advantageous. Eddy current vector can, therefore, be examined in the core region using the local αβγ-coordinate system of a single lamination. The α and β directions are tangential to lamination surfaces, and γ direction is perpendicular to it. Thus, the sum of the normal component of magnetic induction ${\vec{B}}_{\gamma }$ and the tangential component of the magnetic induction ${\vec{B}}_{\alpha \beta }$ give the magnetic induction vector:

(7) $$\vec{B}={\vec{B}}_{\alpha \beta }+{\vec{B}}_{\gamma }$$

**Figure 3:**
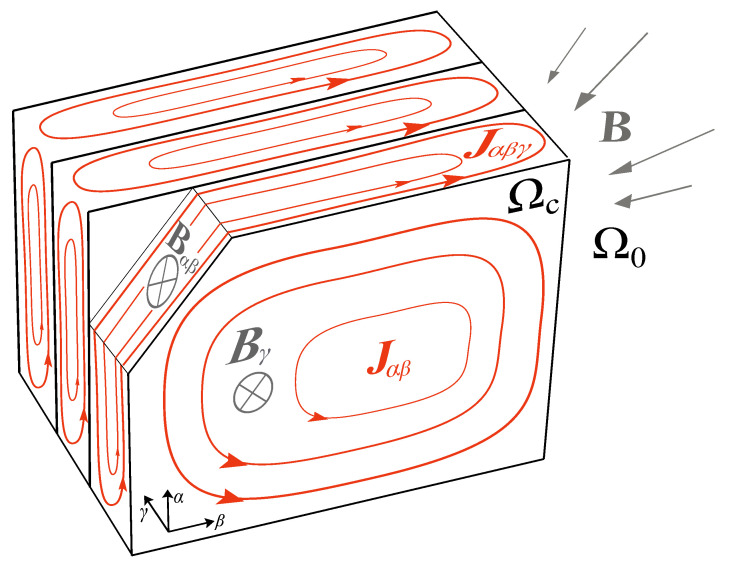
Representation of relevant fields and eddy currents in a simple laminated medium.

This is represented in Figure 3.

Similarly, total current density $\vec{J}$ can be separated into current density of narrow eddy current loops induced by the tangential component of the magnetic induction ${\vec{J}}_{\alpha \beta \gamma }$, and current density of large eddy current loops induced by the normal component of the magnetic induction ${\vec{J}}_{\alpha \beta }$:

(8) $$\vec{J}={\vec{J}}_{\alpha \beta \gamma }+{\vec{J}}_{\gamma }$$

Maxwell Equation (1) for the core region can then be written for each component, as each component induces eddy currents inside the laminations:

(9) $$\nabla \times \rho {\vec{J}}_{\alpha \beta \gamma }=-j\omega {\vec{B}}_{\alpha \beta }$$

(10) $$\nabla \times \rho {\vec{J}}_{\alpha \beta }=-j\omega {\vec{B}}_{\gamma }$$

The total losses due to eddy currents *P* can be calculated with equation:

(11) $$\vec{P}={\vec{P}}_{\alpha \beta \gamma }+{\vec{P}}_{\alpha \beta }$$

where ${\vec{P}}_{\alpha \beta \gamma }$ are the eddy current losses due to eddy currents ${\vec{J}}_{\alpha \beta \gamma }$, and ${\vec{P}}_{\alpha \beta }$ are the eddy current losses due to eddy currents ${\vec{J}}_{\alpha \beta }$. This disassembly of the losses is possible due to orthogonality of ${\vec{J}}_{\alpha \beta \gamma }$ and ${\vec{J}}_{\alpha \beta }$ which is true when the thickness of each lamination is smaller than its width and height since then it is valid:

(12) $$\int_{\Omega_{L}}{\vec{J}}_{\alpha ,\beta }\cdot {\vec{J}}_{\alpha \beta \gamma }dV\approx 0$$

where $\Omega_{L}$ denotes the region of one lamination sheet.

Different approaches are used for the determination of current densities of narrow eddy current loops induced by the tangential component of the magnetic induction and current densities of large eddy current loops induced by the normal component of the magnetic induction. This is due to the fact that because the value of magnetic permeability of the core is significantly lower in the perpendicular than in the tangential direction, ${\vec{J}}_{\alpha \beta \gamma }$ has a negligible effect on ${\vec{J}}_{\alpha \beta }$ and can be ignored when calculating ${\vec{J}}_{\alpha \beta }$. The opposite is untrue.

Thin insulating layers are sandwiched between the thin laminations that make up the core. In terms of magnetic permeability and electrical conductivity, this indicates that the core region is heterogeneous. The quickly varying material properties result in a highly oscillatory spatial dependency of the narrow loops of eddy current density. As a result, it is also necessary to utilize a mesh that is incredibly dense. With the aid of homogenization, this can be prevented. As a result, in order to apply a coarse mesh, the material properties in the core region must be homogenized. This homogenization is carried out in accordance with the average magnetic circuits principle, which results in a new equivalent material with anisotropic material properties ρ and ν, according to [23]. The eddy current density of narrow loops cannot be averaged, but it can be described using magnetic induction, which can be averaged within a coarse mesh’s finite element. The imaginary magnetic reluctivity can be used to account for the weak formulation’s contribution of the eddy current density of narrow loops. As a result, the magnetic reluctivity is expressed as a complex number that has both real and imaginary components. Individual components of the diagonal tensors of electrical resistivity ρ and magnetic reluctivity ν are calculated analytically using equations [24]:

(13) $$\begin{array}{cc} \; & \rho_{\alpha }=\rho_{\alpha }=\rho K_{f}\\ \; & \rho_{\gamma }^{-1}\approx 0\\ \; & \nu_{\alpha }^{-1}=\nu_{\alpha }^{-1}=\nu^{-1}K_{f}+\nu_{0}^{-1}\left(1-K_{f}\right)\\ \; & v_{\gamma }=\nu K_{f}+\nu_{0}\left(1-K_{f}\right) \end{array}$$

where $K_{f}$ denotes the filling factor a laminated core. The diagonal tensors that define the characteristics of the new, substitute material can then be written as:

(14) $$\rho =\left[\begin{array}{ccc} \rho_{\alpha } & \rho_{\beta } & \rho_{\gamma } \end{array}\right]$$

(15) $$\nu =\left[\begin{array}{ccc} \nu_{\alpha } & \nu_{\beta } & \nu_{\gamma } \end{array}\right]+j\left[\begin{array}{ccc} 0 & \kappa & \kappa \end{array}\right]$$

where parameter κ is determined using analytical approach in the preprocessing phase. Parameter κ is calculated using expression:

(16) $$\kappa =\frac{1}{12}\frac{t^{2}\omega }{\rho }$$

where *t* denotes the thickness of the laminations.

When the described procedure of homogenization is used on the core region, with the density vector of narrow eddy current loops being taken into account indirectly, instead of Maxwell’s Equations (1) and (2) the following expressions are used:

(17) $$\begin{array}{cc} \; & \nabla \times \rho {\vec{J}}_{\alpha \beta }=-j\omega \vec{B}\\ \; & \nabla \times v\vec{B}={\vec{J}}_{\alpha \beta } \end{array}$$

In this paper, a formulation based on the magnetic vector potential $\vec{A}$ and the current vector potential $\vec{T}$ is used for the calculation of eddy current losses [27]. Equations (3) and (4) enable the use of expressions:

(18) $$\begin{array}{cc} \; & \vec{B}=\nabla \times \vec{A}\\ \; & {\vec{J}}_{\alpha \beta }=\nabla \times \vec{T} \end{array}$$

Instead of using $\vec{T}$, to attain a symmetric system of equations, time-primitive potential $\vec{\tau }$ is used. Expression associating the two is:

(19) $$\vec{T}=\partial_{t}\vec{\tau }$$

Time-primitive potential $\vec{\tau }$ is interpolated by edge elements [25]. Current vector potential $\vec{A}$ is also interpolated by edge elements $\vec{N_{k}}$. $\vec{A}$ is used in order to strongly ensure the continuity of the normal component of magnetic induction at the core/air interface. Hence, expressions in (17) become:

(20) $$\begin{array}{cc} \; & -j\omega \nabla \times \rho \nabla \times \vec{\tau }+j\omega \nabla \times \vec{A}=0\\ \; & \nabla \times \nu \nabla \times \vec{A}+j\omega \nabla \times \vec{\tau }=0 \end{array}$$

Similarly, in the united air and winding region Equation (5) can be rewritten as:

(21) $$\nabla \times \nu \nabla \times \vec{A}=\nabla \times {\vec{T}}_{s}$$

Then, using interpolation functions $\vec{N_{k}}$ as weighting functions in expressions (20) and (21), the weak AτA-formulation is obtained:

(22) $$\begin{array}{cc} \; & \int_{\Omega_{n}}\nu_{0}\nabla \times \vec{A}\cdot \nabla \times {\vec{N}}_{k}d\Omega +\int_{\Omega_{c}}\nu \nabla \times \vec{A}\cdot \nabla \times {\vec{N}}_{k}d\Omega +\\ \; & \int_{\Omega_{c}}\nabla \times j\omega \vec{\tau }\cdot {\vec{N}}_{k}d\Omega =\int_{\Omega }{\vec{T}}_{0}\cdot \nabla \times {\vec{N}}_{k}d\Omega \\ \; & \int_{\Omega_{c}}j\omega \vec{A}\cdot \nabla \times {\vec{N}}_{k}dV-\int_{\Omega_{c}}\rho \nabla \times j\omega \vec{\tau }\cdot \nabla \times {\vec{N}}_{k}dV=0 \end{array}$$

where the index *k* represents the *k*-th degree of freedom in the finite element [26].

#### Redundant Degrees of Freedom Elimination

It is possible to eliminate the redundant degrees of freedom. Only the γ component of $\vec{\tau }$ is required when computing the eddy current density of large loops using the local αβγ-coordinate system for each lamination, where the γ-direction represents the normal direction of the lamination sheet. The tangential component of $\vec{\tau }$ that is in αβ directions is, therefore, redundant. This implies that it is possible to eliminate it from the eddy current losses calculation. This can be accomplished by building a structural mesh inside the core, such that each edge of each finite element is either parallel to or perpendicular to the γ-direction. Then, the tangential an normal components of $\vec{\tau }$ can be determined using edge degrees of freedom ${\vec{\tau }}_{k}$, with the following expression connecting the two:

(23) $$\vec{\tau }=\sum \tau_{k}{\vec{N}}_{k}$$

**Figure 4:**
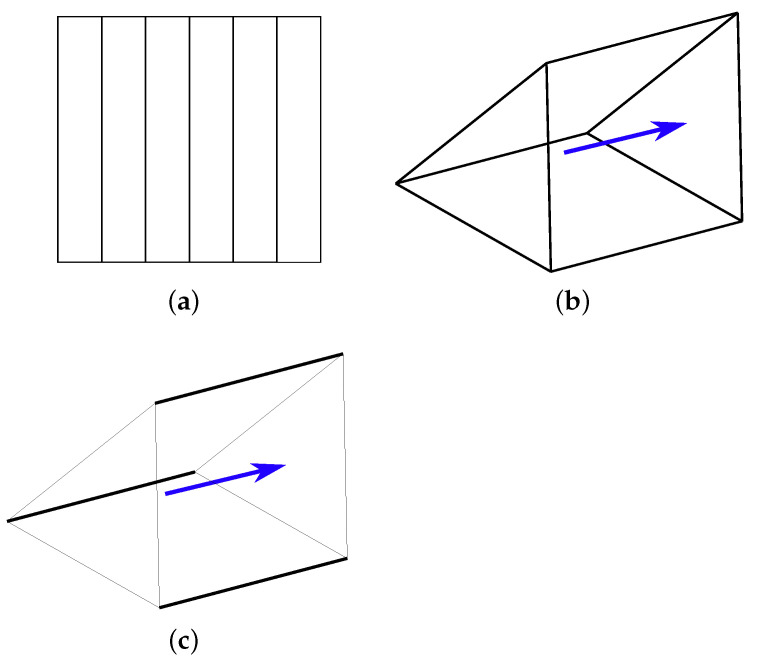
(**a**) Cross-section of a laminated core; (**b**) a finite element with 9 edges that are either parallel or perpendicular to the anisotropy vector depicted by a blue arrow; and (**c**) a finite element without edges to which redundant degrees of freedom are attached.

Then, for each edge of each finite element coarse mesh in the core region a rule is obtained:

(24) $${\vec{N}}_{k}\cdot {\vec{a}}_{\gamma }=\left\{\begin{array}{cc} 0, & \;\mathrm{then}\;\tau_{k}=0\\ \left|N_{k}\right|, & \;\mathrm{then}\;\tau_{k}=\tau_{k} \end{array}\right.$$

where vector ${\vec{a}}_{\gamma }$ is the anisotropy vector. Obtained edge elements are shown in Figure 4. Using (24), the redundant degrees of freedom from the matrix of coefficients are eliminated in the preprocessing phase. This allows the improvement of the convergence speed when calculating the eddy current losses.

## 3. Results and Discussion

**Figure 5:**
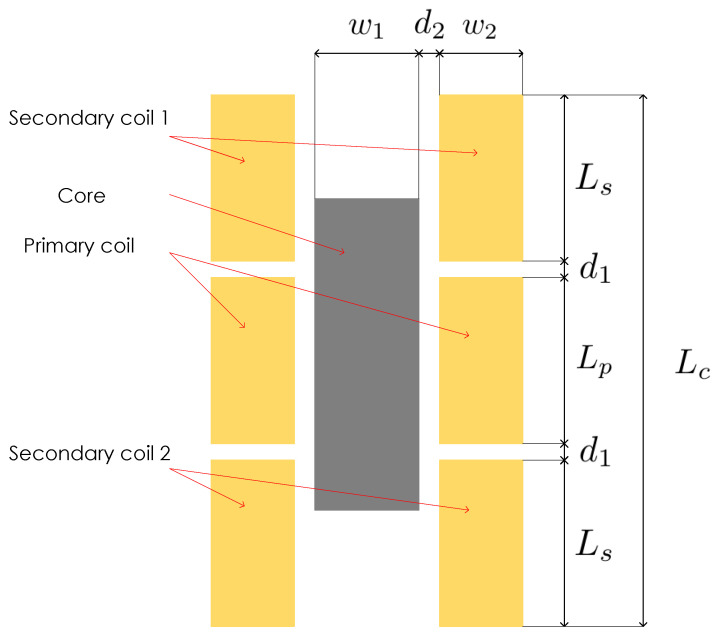
LVDT design for simulation.

A 3D model of a LVDT is used for the analysis of the proposed methodology for calculation of eddy current losses. A weak AτA-formulation, described in Section 2.2 of Section 2, is used to calculate the eddy current losses in LVDT model. The LVDT model corresponds to the one shown in Figure 5, with the parameters and their physical values listed in Table 1. The geometrical dimensions are merely indicative. Since the analysis was performed for different frequencies and materials, those parameters are listed in Table 2.

Firstly, in order to validate the proposed methodology that uses a weak AτA-formulation with proposed improved elimination of redundant degrees of freedom, a comparison of two types of FEM simulations was performed. One simulation was completed with a novel approach, and the other was completed by using the typical weak AτA-formulation. The second approach was already validated in [26]. An iterative technique based on the CG algorithm is used to solve the linear system arising from the formulation in the simulations. Both simulations were run on the same mesh. The simulations were run on the LVDT model with core centred at 0 mm (without displacement), for the core material of relative permeability 10,000 and conductivity of core $2\times 10^{6}$ S/m, at frequency of 50 Hz. The amount of total eddy current losses is equal in both cases and amounts to 5.2 mW. However, due to the elimination of redundant edge elements, there was a major difference in calculation time. Since the current vector potential is used as the excitation, no direct modelling of the coil is required. This is why it is possible to use the same mesh for all positions and the core position for the same number of mesh elements does not significantly affect the duration of the simulation for different core positions. The number of elements of the core mesh is 46,800, while the number of elements in the air is 401,573. For comparison purposes, the same mesh was used for both formulations. While the simulation for a single position that used the typical AτA-formulation ran for 6400 s, the simulation using the proposed approach ran for 2586 s, thus improving the time 2.47 times which is a significant improvement.

**Figure 6:**
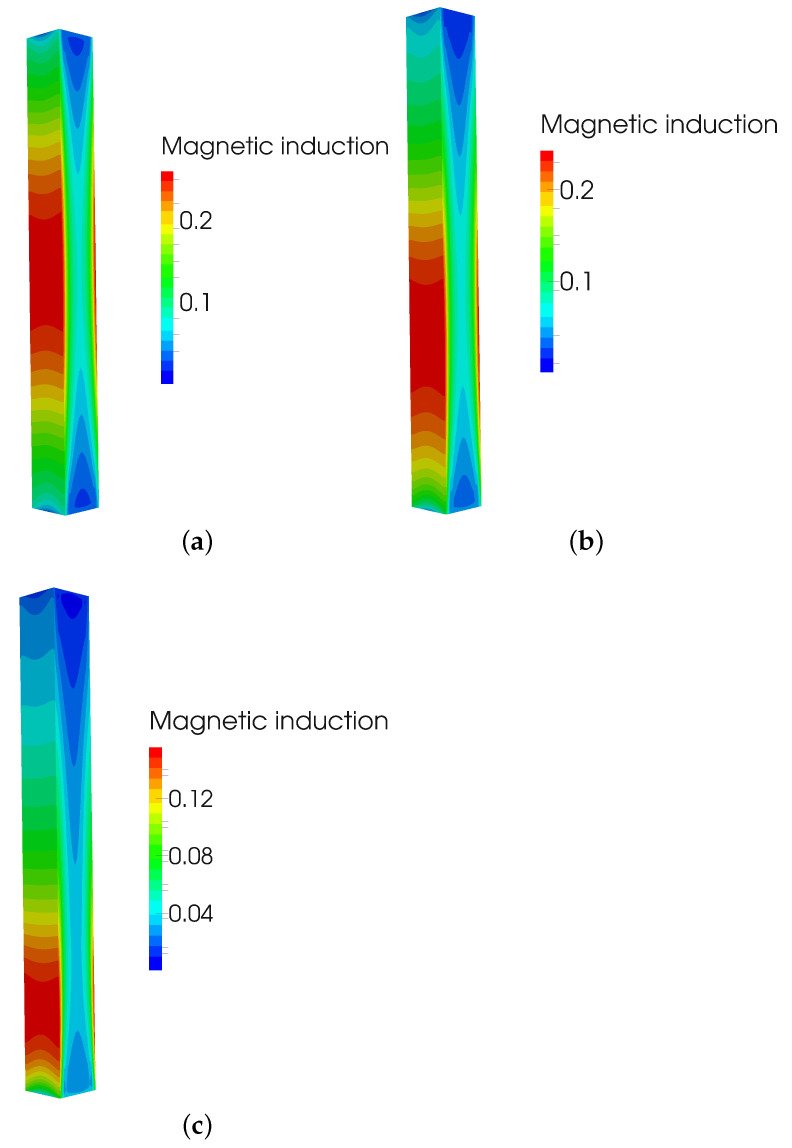
(**a**) Magnetic induction at position 1 = 0 mm; (**b**) Magnetic induction at position 2 = 20 mm; and (**c**) Magnetic induction at position 3 = 50 mm.

**Figure 7:**
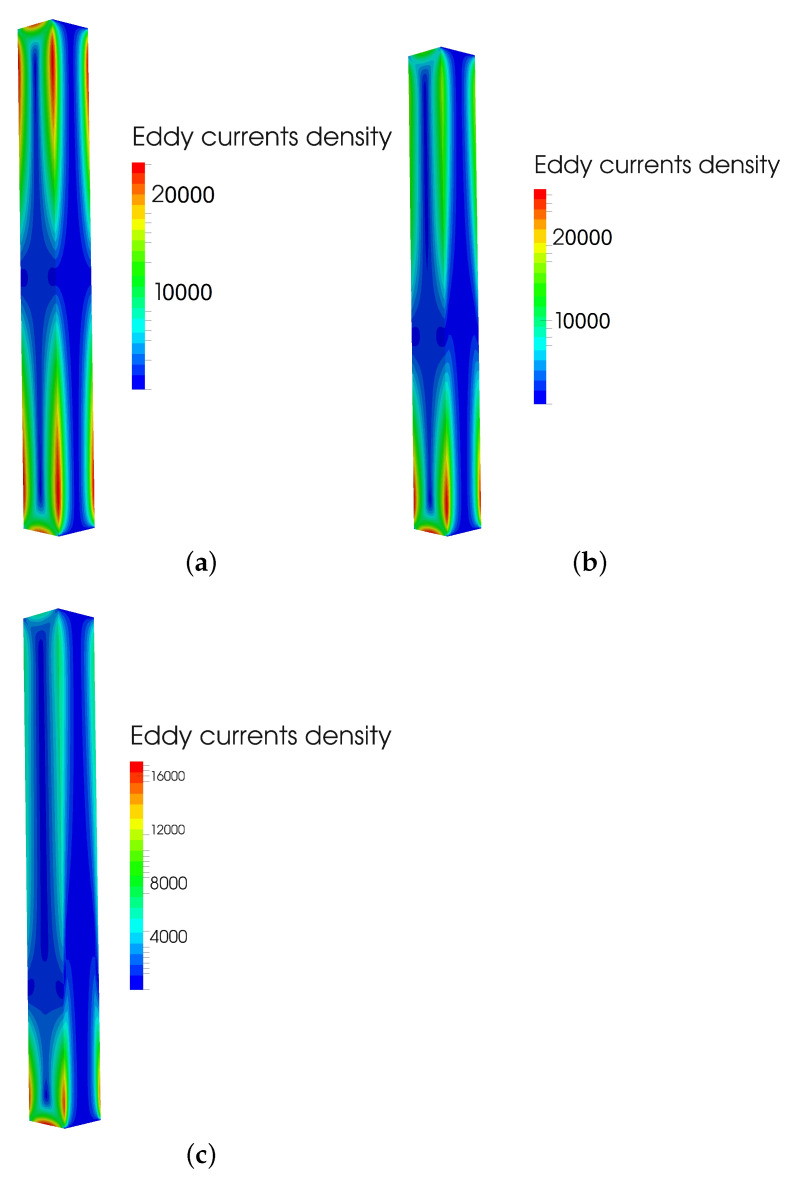
(**a**) Eddy current density at position 1 = 0 mm; (**b**) Eddy current density at position 2 = 20; and (**c**) Eddy current density at position 3 = 50 mm.

After that, multiple simulations of eddy current losses based on the core displacement were made. Figure 6 presents magnetic induction at frequency of 50 Hz, for the core material of relative permeability 10,000 and conductivity of core $2\times 10^{6}$ S/m at three positions, position 1 of core at 0 mm without displacement, position 2 in which core is displaced by 20 mm, and position 3 with the displacement of 50 mm. Similarly, for the same positions, material, and frequency, Figure 7 presents eddy current density. Eddy currents are, therefore, most influential at position without core displacement, as the magnetic induction is then at its highest.

**Figure 8:**
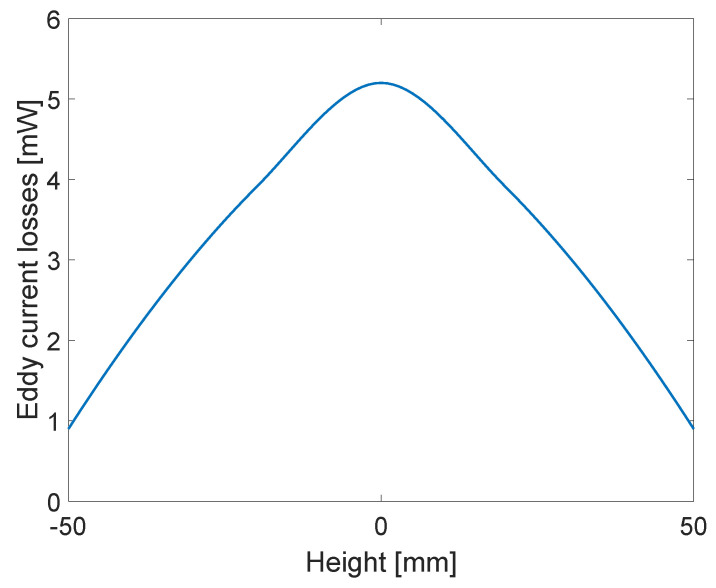
Eddy current losses dependence on position.

Then, the core was moved from position −50 mm to 50 mm, at 10 mm intervals, with the position of centre where there is no displacement at 0 mm. For those positions, eddy current losses were calculated for material properties of relative permeability 10,000 and conductivity of core $2\times 10^{6}$ S/m, at frequency of 50 Hz. The results are presented in Figure 8, with interpolation performed for other positions. Not only can the influence of eddy currents be seen from Figure 6, like in previous research, but the presented methodology has enabled the quantification of those losses, as seen in Figure 8.

**Figure 9:**
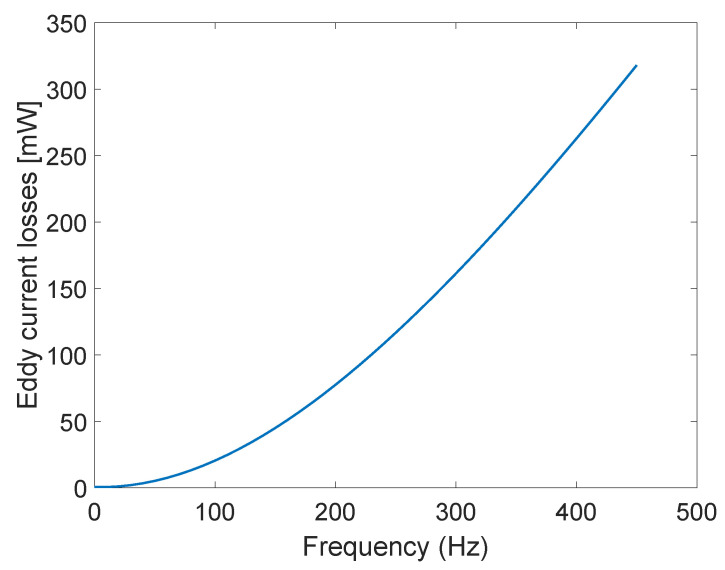
Eddy current losses dependence on frequency.

Eddy current losses were also calculated at position of the laminated core at 0 mm without displacement, for the core material of relative permeability 10,000 and conductivity of core $2\times 10^{6}$ S/m, at frequencies ranging from 50 Hz to 450 Hz with the step of 100 Hz. Interpolated results are shown in Figure 9. As expected, eddy current losses rise with frequency. Presented methodology enables the designer of LVDT to calculate the losses at wanted working frequency and then determine whether to modify the LVDT core properties, i.e., change the core material or increase the number of laminations in order to lower the losses and thus increase reliability.

**Figure 10:**
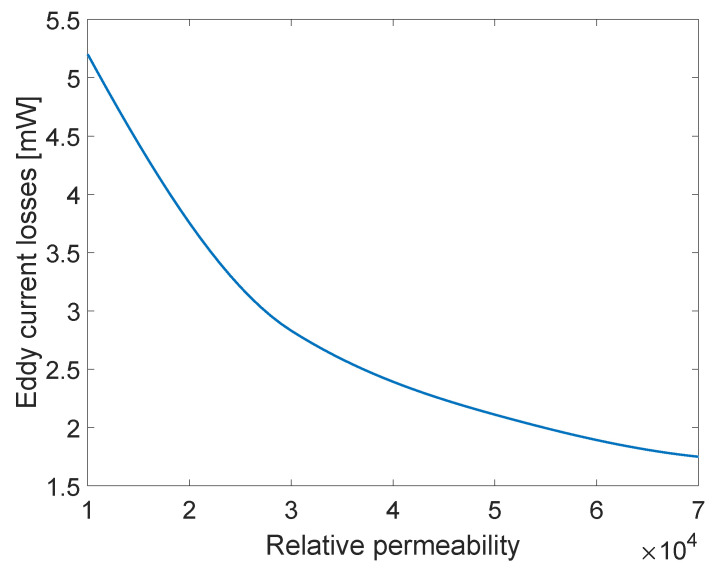
Eddy current losses dependence on relative permeability of the core magnetic material.

An analysis of eddy current losses for different core materials of a laminated core was also made. Materials of relative permeability of 10,000, 30,000, 50,000, and 70,000 were used. Corresponding core conductivity was always set to $2\times 10^{6}$ S/m. The results are presented in Figure 10. The losses decrease with the increase in relative permeability, as is expected. Since permeability is directly linked to the performance and reliability of the device [18], and some materials are in certain periods not available, it is important to be able to obtain knowledge of eddy current losses for a range different materials.

Results presented in this paper, along with the conclusions established in [5,18] about the influence of eddy currents on the sensor performance, can be used to develop a set of limitations and standards in LVDT design dependent on eddy current losses with the goal of sensor optimization. Methodology for the calculation of eddy current losses in LVDTs presented in this paper can also be used for modified designs of LVDTs, different materials and different frequencies, depending on the final product requirements.

## 4. Conclusions

LVDTs are regularly used magnetic displacement sensors due to their high precision and robust design. Due to the open-type core a typical LVDT has, depending on the core material, it is very important to take eddy currents into the account. This is especially important if steel materials are used. It is important to take into consideration the magnetic permeabilities and electrical conductivities of magnetic materials in the design of magnetic displacement sensors. The frequency at which the sensor operates should also be considered when choosing the core materials due to the increase in eddy current losses with frequency.

In this paper, the presented approach for eddy current losses calculation using a weak AτA-formulation has taken into the account the particularity of the geometry of the LVDT core. Numerical homogenization offers a simple way of taking into account edge effects for eddy currents induced by the magnetic flux tangential to the lamination surfaces. The results are in good accord with other potential approaches. The suggested method, which produces precise results, is simple to utilize for LVDT design optimization. Analysis of the results obtained using presented methodology for eddy current losses calculation in LVDTs enables the designer of those devices to know the losses depending on frequency, material, and number of laminations and, thus, optimize the design of the LVDT. That means that the presented methodology could influence the improvement of design standards for LVDTs, as well as other electromagnetic devices. The simulation results take into account the standard LVDT design, but the methodology can also be implemented for the analysis of modified LVDT designs.

The proposed eddy current losses methodology’s scientific goal is to provide a tool that can be used to study the eddy current phenomenon that occurs in LVDTs. Further work could be based on the development of improved standardized design instructions dependant on the core loss ratio of LVDTs dependent on frequency, material characteristic, material availability, and necessary dimensions of the designed sensor.

## Figures and Tables

**Table 1:** Design parameters used for LVDT analysis.

Parameter	Value
Primary coil length—$L_{p}$ [mm]	60
Primary coil width—$w_{2}$ [mm]	20
Secondary coil length—$L_{s}$ [mm]	60
Secondary coil width—$w_{2}$ [mm]	20
Core length [mm]—$L_{c}$	100
Core width [mm]—$w_{1}$	10
Distance between coils [mm]—$d_{1}$	2
Distance between core and coils—$d_{2}$ [mm]	5
No. of coil turns	1000

**Table 2:** Material parameters used for LVDT analysis.

Parameter	Value	Variation
Supply frequency [Hz]	50	50–400
Relative permeability of core	10,000	10,000–70,000
Conductivity of core [S/m]	$2\times 10^{6}$	$2\times 10^{6}$

## Data Availability

Not applicable.

## Abbreviations

The following abbreviations are used in this manuscript:

LVDT	Linear variable differential transformer
FEM	Finite element method

## References

[1] Masi A., Danisi A., Losito R., Martino M., Spiezia G. (2011). Study of magnetic interference on an LVDT: FEM modeling and experimental measurements. J. Sens..

[2] Mirzaei M., Machac J., Ripka P., Chirtsov A., Vyhnanek J., Grim V. (2020). Design of a flat-type magnetic position sensor using a finite-difference method. IET Sci. Meas. Technol..

[3] Lu X., Tian G., Wang Z., Li W., Yang D., Li H., Wang Y., Ni J., Zhang Y. (2022). Research on the Time Drift Stability of Differential Inductive Displacement Sensors with Frequency Output. Sensors.

[4] Hoxha A., Passarotto M., Qama G., Specogna R. (2022). Design Optimization of PCB-Based Rotary-Inductive Position Sensors. Sensors.

[5] He Q., Fan S., Chen N., Tan R., Chen F., Fan D. (2021). Analysis of Inductive Displacement Sensors with Large Range and Nanoscale Resolution. Appl. Sci..

[6] Sykulski J.K., Sykulska E., Hughes S.T. (1992). Application of finite-element modelling in LVDT-design. Int. J. Comput. Math. Electr. Electron. Eng..

[7] Yang Y.-S., Bae K.-Y. (2021). Finite Element Analysis of the Effects of Process and Material Parameters on the LVDT Output Characteristics. Korean Soc. Manuf. Process Eng..

[8] Ripka P., Mirzaei M., Blažek J. (2022). Magnetic position sensors. Meas. Sci. Technol..

[9] Ara K. (1972). A differential transformer with temperature and excitation independent output. IEEE Trans. Instrum. Meas..

[10] Martino M., Danisi A., Losito R., Masi A., Spiezia G. (2010). Design of a Linear Variable Differential Trans-former with High Rejection to External Interfering Magnetic Field. IEEE Trans. Magn..

[11] Midgley G.W., Howe D., Mellor P.H., Nicolet A., Belmans R. (1995). Improved Linearity Linear Variable Differential Transformers (LVDTs) Through the Use of Alternative Magnetic Materials. Electric and Magnetic Fields.

[12] Cao J., Wei M., Zhou D. (2022). Experimental Study on the Coupling Mechanism of Sensors under a Strong Electromagnetic Pulse. Sensors.

[13] Rerkratn A., Tongcharoen J., Petchmaneelumka W., Riewruja V. (2022). Linear-Range Extension for Linear Variable Differential Transformer Using Hyperbolic Sine Function. Sensors.

[14] Vishal D., Ramesh T.K., Pardhu V., Naga C. Design of LVDT for Aeronautical Jet Engines. Proceedings of the IEEE International Conference on RTEICT.

[15] Saurav S., Muthuganesh M., Chaurasia P.K., Murugan S. (2019). Analysis, Design, and Development of a Compact LVDT for In-Reactor Experiments. IETE J. Educ..

[16] Mandal H., Bera S.K., Saha S., Sadhu P.K., Bera S.C. (2018). Study of a Modified LVDT Type Displacement Transducer With Unlimited Range. IEEE Sens. J..

[17] Petchmaneelumka W., Rerkratn A., Luangpol A., Riewruja V. (2018). Compensation of temperature effect for LVDT transducer. J. Circuits Syst. Comput..

[18] Yañez-Valdez R., Alva-Gallegos R., Caballero-Ruiz A., Ruiz-Huerta L. (2012). Selection of Soft Magnetic Core Materials Used on an LVDT Prototype. J. Appl. Res. Technol..

[19] Chiriac H., Hristoforou E., Neagu M., Peptanariu M., Castano F.J. (2000). Linear variable differential trans-former sensor using Fe-rich amorphous wires as an active core. J. Appl. Phys..

[20] Chiriac H., Hristoforou E., Neagu M., Peptanariu M. (2000). Linear variable differential transformer sensor us-ing glass-covered amorphous wires as active core. J. Magn. Magn. Mater..

[21] Tian G.Y., Zhao Z.X., Baines R.W. (1997). Computational algorithms for linear variable differential transform-ers (LVDTs). IEE Proc. Sci. Meas. Technol..

[22] Hollaus K., Schoberl J. (2018). Some 2-D Multiscale Finite-Element Formulations for the Eddy Current Problem in Iron Laminates. IEEE Trans. Magn..

[23] Kaimori H., Kameari A., Fujiwara K. (2007). FEM Computation of Magnetic Field and Iron Loss in Laminated Iron Core Using Homogenization Method. IEEE Trans. Magn..

[24] De Gersem H., Vanaverbeke S., Samaey G. (2012). Three-Dimensional–Two-Dimensional Coupled Model for Eddy Currents in Laminated Iron Cores. IEEE Trans. Magn..

[25] Bíró O. (1999). Edge element formulations of eddy current problems. Comput. Methods Appl. Mech. Eng..

[26] Frljic S., Trkulja B., Žiger I. (2021). Calculation of the Eddy Current Losses in a Laminated Open-Type Trans-former Core Based on the A,T-A Formulation. Appl. Sci..

[27] Frljic S., Trkulja B. (2021). Two-Step Method for the Calculation of Eddy Current Losses in an Open-Core Transformer. IEEE Trans. Magn..

[28] Hanser V., Schobinger M., Hollaus K. SEfficient Computation of Eddy Current Losses in Laminated Cores with Air Gaps by the Multiscale FEM. Proceedings of the 2022 23rd International Conference on the Computation of Electromagnetic Fields (COMPUMAG).

